# Supplementary material for: Stochastic disturbance regimes alter patterns of ecosystem variability and recovery
Source: PLoS One. 2020 Mar 9;15(3):e0229927. doi: 10.1371/journal.pone.0229927 (PMC7062255; doi:10.1371/journal.pone.0229927)
Supplement: S1 Table — (DOCX) [file pone.0229927.s001.docx]

**Table S1 Parameters and results for the deterministic and stochastic simulations used to validate the model and explore ecosystem variability and recovery dynamics.** Individual simulations were run for 100 time steps on a 100 x 100 grid. Disturbed sites recover through succession, achieving full recovery when they reach successional stage 8. For stochastic parameters, values shown are the mean ± standard deviation for the duration of the simulation. The resulting mean and variance of the proportion of the landscape covered by the mature successional stage produced by each simulation are given in the last two columns. See Figure S1 for additional results from these simulations.

| Simulation type | Disturbance spatial extent  (length x width) | Disturbance return interval  (time steps) | Disturbance severity (successional stage to which a cell is reset following a disturbance event) | Mean proportion of the landscape covered by the mature stage  (%) | Variance of the proportion of the landscape covered by the mature stage (*Vp*) |
| --- | --- | --- | --- | --- | --- |
| Deterministic | 35 x 35 | 8 | 1 | 89.3 | 4.14 |
| Stochastic size | 35.5 x 35.5 ± 20.2 | 8 | 1 | 90.8 | 9.54 |
| Stochastic frequency | 35 x 35 | 8 ± 7.9 | 1 | 89.1 | 10.28 |
| Stochastic severity | 35 x 35 | 8 | 3.5 ± 2.0 | 93.9 | 6.15 |
| Fully stochastic | 35.5 x 35.5 ± 20.2 | 8 ± 7.9 | 3.5 ± 2.0 | 91.3 | 13.28 |
