# Supplementary material for: Stochastic disturbance regimes alter patterns of ecosystem variability and recovery
Source: PLoS One. 2020 Mar 9;15(3):e0229927. doi: 10.1371/journal.pone.0229927 (PMC7062255; doi:10.1371/journal.pone.0229927)
Supplement: S2 Table — (DOCX) [file pone.0229927.s002.docx]

**Table S2** **Spatial (S) and temporal (T) scales of disturbance used to create state-space diagrams illustrating potential disturbance dynamics.** We executed deterministic and stochastic versions of the model covering the full range of these S and T values. Landscape size is constant at 10,000 units and recovery time is constant at 8.

| ***Spatial scale of disturbance events*** | | | |  |
| --- | --- | --- | --- | --- |
| Disturbance extent  (number of cells) | | $\frac{Disturbance extent}{Landscape extent}$ | |  |
|  | | (% of landscape) | |  |
| 10 x 10 | | 1 | |  |
| 50 x 50 | | 25 | |  |
| 71 x 71 | | 50 | |  |
| 86 x 86 | | 74 | |  |
| 100 x 100 | | 100 | |  |
| ***Temporal scale of disturbance events*** | | | | |
| Disturbance return interval  (time steps) | Disturbance frequency  (per time step during simulation) | | $\frac{\begin{aligned} Disturbance return \\ \mathrm{interval} \end{aligned}}{\begin{aligned} Ecosystem recovery \\ \mathrm{time} \end{aligned}}$ | |
| 80 | 0.0125 | | 10 | |
| 40 | 0.025 | | 5 | |
| 8 | 0.125 | | 1 | |
| 4 | 0.25 | | 0.5 | |
| 0.8 | 1.25 | | 0.1 | |
| 0.4 | 2.5 | | 0.05 | |
| 0.08 | 12.5 | | 0.01 | |
