# Supplementary material for: Stochastic disturbance regimes alter patterns of ecosystem variability and recovery
Source: PLoS One. 2020 Mar 9;15(3):e0229927. doi: 10.1371/journal.pone.0229927 (PMC7062255; doi:10.1371/journal.pone.0229927)
Supplement: S3 Table — (DOCX) [file pone.0229927.s003.docx]

**Table S3 Empirical studies evaluated for data-model agreement.** Studies were located through electronic searches of the Web of Science (Clarivate Analytics) citation index using the keywords: disturbance, heterogeneity, stability, variance and variability, and by examining the references in these studies. We selected studies that focused on structural (cover, abundance) or compositional responses to disturbance and reported values of mean and variance to align with the type of responses simulated by the model. The reported result was used to classify the ecosystem dynamics† of each study as indicated parenthetically. Deterministic and stochastic models were parameterized using the values for disturbance frequency and size reported in each study. Values shown in the last five columns are variance of occupancy of the mature successional stage (*Vp*) for the duration of 100,000 simulations under a given model configuration. These values together with the mean were used to characterize predicted ecosystem dynamics† as indicated parenthetically: Table section 1 shows cases where a stochastic model configuration outperformed the deterministic configuration, resulting in agreement between empirical results and predictions, i.e., where the observed dynamics qualitatively matched the dynamics predicted by a stochastic model parameterized with same the disturbance characteristics. To facilitate comparison among studies, we also computed the scaled disturbance parameters, T (disturbance return interval divided by ecosystem recovery interval) and S (disturbance extent divided by landscape extent, as a proportion). Studies marked with an asterisk were not included in the Fraterrigo and Rusak (2008) review.

| **Reference** | **Ecosystem** | **Disturbance** | **Response variable** | **Empirical result** | **T** | **S** | **Deter-ministic** | **Stochas Freq** | **Stochas**  **Extent** | **Stochas**  **Severity** | **Fully**  **Stochas** |
| --- | --- | --- | --- | --- | --- | --- | --- | --- | --- | --- | --- |
| 1. *Responses correctly classified by one or more stochastic model configurations; misclassified by deterministic model* | | | | | | | | | | | |
| Coleman, Underwood [1] | Marine | Release from grazing | Rocky intertidal algal cover and biomass | High variance (C/E) | 0.3 | 0.5 | 8.87 (D) | 24.8 (E) | 27.9 (E) | 21.3 (E) | 36.6 (E) |
| Collins [2] | Grassland | Fire, high frequency | Grasshopper community composition | High variance  (C/E) | 0.33 | 1 | 0.316 (F) | 25.6 (E) | 21.0 (E) | 12.5 (C) | 37.4 (E) |
| Houseman, Mittelbach [3]* | Grassland | Biomass reduction and fertilization | Plant community composition | High variance  (C/E) | 0.28 | 0.5 | 8.94 (D) | 22.7 (E) | 26.2 (E) | 20.5 (E) | 35.8 (E) |
| Hsieh, Reiss [4] | Marine | Commercial fish harvest | Fish population abundance | High variability  (C/E) | 0.5 | 1 | 0.316 (F) | 38.0 (E) | 30.4 (E) | 36.6 (E) | 41.5 (E) |
| Molinos and Donohue [5]* | Freshwater | Sediment pulses | Benthic invertebrate abundance and richness | High variance  (C/E) | 0.10 | 0.5 | 0.749 (F) | 3.76 (F) | 3.05 (F) | 1.43 (F) | 12.3 (C) |
| Queiroz, Lima [6] | Marine | Oil spill | Intertidal macroinvertebrate abundance | Equilibr.  (A) | 100 | 1 | 9.31 (B) | 9.27 (B) | 6.50 (B) | 6.89 (B) | 4.52 (A) |
| Rusak, Yan [7] | Freshwater | Predator manipulation | Zooplankton abundance and richness | High variance  (C/E) | 0.15 | 1 | 0.316 (F) | 5.73 (D) | 13.6 (C) | 1.97 (F) | 16.5 (C) |
| Ryan, Mcmanus [8]* | Freshwater | Saline incursion | Phytoplankton abundance | Low variance  (B) | 18.25 | 0.5 | 10.7 (C) | 9.70 (B) | 11.2 (C) | 7.30 (B) | 9.11 (B) |
| Stark, Riddle [9] | Marine | Sewage discharge | Benthic community composition | Low variance  (D) | 0.08 | 0.5 | 0.455 (F) | 2.19 (F) | 1.59 (F) | 0.690 (F) | 7.68 (D) |
| Vargas [10]* | Forest | Hurricane | Gross primary production | High variance  (C/E) | 0.5 | 1 | 0.316 (F) | 37.9 (E) | 30.4 (E) | 36.8 (E) | 41.6 (E) |
| Warwick and Clarke [11] | Marine | Drilling and mining | Meio- and macrobenthic  abundance | High variance  (C/E) | 0.2 | 0.5 | 6.24 (D) | 16.4 (C) | 19.9 (C) | 14.7 (C) | - 1. (E) |
| 1. *Responses correctly classified by both deterministic and stochastic models* | | | | | | | | | | | |
| Arkle, Pilliod [12] | Freshwater | Fire | Macroinvertebrate community composition | High variance  (C/E) | 8.00 | 0.46 | 14.4 (C) | 14.0 (C) | 16.6 (C) | 10.4 (C) | 11.7 (C) |
| Bêche and Resh [13]* | Freshwater | Drought | Macroinverte-brate community composition | High variance  (C/E) | 12.0 | 1 | 26.0 (E) | 26.1 (E) | 17.1 (C) | 19.6 (C) | 12.8 (C) |
| Bertocci, Maggi [14] | Marine | Biomass removal, low intensity | Benthic intertidal community composition | High variance  (C/E) | 1.00 | 1 | 33.1 (E) | 49.3 (E) | 38.2 (E) | 49.8 (E) | 38.7 (E) |
| Bertocci, Maggi [14] | Marine | Biomass removal, medium intensity | Benthic intertidal community composition | High variance  (C/E) | 1.50 | 1 | 49.3 (E) | 49.7 (E) | 38.9 (E) | 45.8 (E) | 34.7 (E) |
| Bertocci, Maggi [14] | Marine | Biomass removal, high intensity | Benthic intertidal community composition | High variance  (C/E) | 3.00 | 1 | 45.5 (E) | 43.4 (E) | 32.1 (E) | 35.8 (E) | 26.5 (E) |
| Brown [15] | Freshwater | Drought and flooding | Macroinvertebrate community composition | High variance  (C/E) | 3.33 | 1 | 44.0 (E) | 42.5 (E) | 32.3 (E) | 33.7 (E) | 25.7 (E) |
| Chapman, Underwood [16] | Marine | Sewage discharge | Encrusting benthos abundance | Equilibr.  (A) | 0.10 | 0.50 | 0.316 (A) | 0.316 (A) | 0.316 (A) | 0.316 (A) | 0.316 (A) |
| Collins [2] | Grassland | Fire, low frequency | Grasshopper community composition | High variance  (C/E) | 6.67 | 1 | 33.8 (E) | 33.0 (E) | 23.2 (E) | 24.8 (E) | 18.6 (C) |
| Collins [2] | Grassland | Fire, medium frequency | Grasshopper community composition | High variance  (C/E) | 1.33 | 1 | 48.1 (E) | 50.0 (E) | 39.5 (E) | 46.9 (E) | 36.1 (E) |
| Collins [2] | Grassland | Fire, low frequency | Plant community composition | High variance  (C/E) | 20 | 1 | 20.5 (C) | 20.2 (C) | 13.8 (C) | 14.7 (C) | 10.9 (C) |
| Collins [2] | Grassland | Fire, medium frequency | Plant community composition | High variance  (C/E) | 4 | 1 | 41.3 (E) | 39.8 (E) | 28.8 (E) | 31.8 (E) | 23.3 (E) |
| Collins [2] | Grassland | Fire, high frequency | Plant community composition | High variance  (C/E) | 1 | 1 | 33.1 (E) | 49.4 (E) | 38.3 (E) | 49.8 (E) | 38.9 (E) |
| Cottingham, Rusak [17] | Freshwater | Eutrophica-tion | Algal pigment concentrations | High variance  (C/E) | 2.00 | 1 | 49.6 (E) | 47.9 (E) | 36.5 (E) | 41.9 (E) | 31.1 (E) |
| Forrest and Arnott [18] | Freshwater | Nutrient enrichment | Zooplankton abundance | High variance  (C/E) | 1.50 | 1 | 49.3 (E) | 49.6 (E) | 39.2 (E) | 46.3 (E) | 34.5 (E) |
| Fuhlendorf, Harrell [19] | Grassland | Fire and grazing | Vegetation cover and structure | High variance  (C/E) | 3.00 | 0.30 | 13.7 (C) | 15.1 (C) | 19.6 (C) | 10.9 (C) | 15.1 (C) |
| Mou, Jones [20] | Forest | Harvest, girdling | Cover of species regenerating from seed bank | High variance  (C/E) | 1.00 | 1 | 33.1 (E) | 49.3 (E) | 38.3 (E) | 49.8 (E) | 38.6 (E) |
| Navarro, Ballesteros [21] | Marine | Large storm | Phytoplankton community composition | High variance  (C/E) | 4.00 | 1 | 41.3 (E) | 41.4 (E) | 28.8 (E) | 31.8 (E) | 22.9 (E) |
| Reed, Raimondi [22] | Marine | El Niño | Kelp forest community abundance | High variance  (C/E) | 1.50 | 1 | 49.3 (E) | 49.7 (E) | 38.8 (E) | 45.9 (E) | 34.4 (E) |
| Stark, Riddle [9] | Marine | Sewage discharge | Soft-sediment benthic abundances | High variance  (C/E) | 1.00 | 0.5 | 16.7 (C) | 32.3 (E) | 33.6 (E) | 25.1 (E) | 30.9 (E) |
| Williams and Baker [23]* | Forest | Fire | Tree density | High variance  (C/E) | 0.68 | 0.39 | 11.1 (C) | 28.2 (E) | 31.3 (E) | 19.8 (E) | 31.4 (E) |
| Williams and Baker [23]* | Forest | Fire | Tree density | High variance  (C/E) | 0.68 | 0.59 | 11.3 (C) | 35.1 (E) | 33.9 (E) | 28.6 (E) | - 1. (E) |
| 1. *Responses correctly classified by deterministic model; misclassified by one or more stochastic models* | | | | | | | | | | | |
| Liu, Xu [24]* | Grassland | Fire and fertilization | Soil microbial biomass | High variance  (C/E) | 13.03 | 0.5 | 12.6 (C) | 12.5 (C) | 13.4 (C) | 9.10 (B) | - 1. (B) |
| 1. *Responses misclassified by all models* | | | | | | | | | | | |
| Forrest and Arnott [18] | Freshwater | Nutrient enrichment | Phytoplanton abundance | Low variance  (B) | 1.50 | 1 | 49.3 (E) | 49.6 (E) | 39.2 (E) | 46.3 (E) | 34.5 (E) |
| Jellyman, Booker [25]* | Freshwater | Flow disturbance | Fish biomass and community composition | High variance  (C/E) | 0.04 | 1 | 0.320 (F) | 0.378 (F) | 0.316 (F) | 0.318 (F) | 0.321 (F) |
| Jiang, Cheng [26]* | Marine | Fish harvest | Fish biomass and community composition | High variance  (C/E) | 0.03 | 1 | 0.316 (F) | 0.316 (F) | 0.316 (F) | 0.316 (F) | 0.316 (F) |
| Jones, Chiu [27] | Freshwater | Typhoon | Bacterioplank-ton/phyto-plankton community composition | Low variance  (B) | 3.75 | 1 | 42.3 (E) | 40.5 (E) | 29.2 (E) | 32.5 (E) | 24.0 (E) |
| Kashian, Turner [28] | Forest | Fire | Stand density | High variance  (C/E) | 100 | 1 | 9.3 (B) | 9.3 (B) | 6.5 (B) | 6.8 (B) | 5.0 (B) |
| Lardicci, Rossi [29] | Marine | Thermal pollution | Meio- and microbenthic abundance | Equilibr.  (A) | 0.02 | 0.25 | 0.318 (F) | 0.331 (F) | 0.320 (F) | 0.317 (F) | 0.317 (F) |
| Micheli, Cottingham [30] | Forest | Predator removal | Small mammal and bird abundance | High variance  (C/E) | 0.005 | 1 | 0.316 (F) | 0.316 (F) | 0.316 (F) | 0.316 (F) | 0.316 (F) |
| Micheli, Cottingham [30] | Freshwater | Exotic planktivore invasion | Zooplankton abundance | High variance  (C/E) | 0.01 | 1 | 0.316 (F) | 0.316 (F) | 0.316 (F) | 0.316 (F) | 0.316 (F) |
| Piazzi, Balata [31] | Marine | Sewage discharge | Epiphyte community composition and population abundance | High variance  (C/E) | 0.003 | 1 | 0.316 (F) | 0.316 (F) | 0.316 (F) | 0.316 (F) | 0.316 (F) |
| Terlizzi, Scuderi [32] | Marine | Sewage discharge | Total molluscan population abundance | High variance  (C/E) | 0.010 | 0.30 | 0.316 (F) | 0.316 (F) | 0.316 (F) | 0.316 (F) | 0.316 (F) |
| Terlizzi, Scuderi [32] | Marine | Sewage discharge | Molluscan community composition | Low variance  (B) | 0.0003 | 0.30 | 0.316 (F) | 0.316 (F) | 0.316 (F) | 0.316 (F) | 0.316 (F) |
| Warwick and Clarke [11] | Marine | Drilling and mining | Reef fish abundance | High variance  (C/E) | 0.025 | 0.5 | 0.316 (F) | 0.316 (F) | .0316 (F) | 0.317 (F) | 0.316 (F) |

**†Notes:** Regions of qualitatively different ecosystem dynamics are based on the mean and variance of the proportion of the landscape occupied by the mature successional stage. These were defined: region A, where the landscape is relatively undisturbed, the mature stage is the dominant cover type (> 50% of the landscape) with very low variance (< 5), indicating equilibrium dynamics; region B, where disturbance is more frequent, the mature stage is the dominant cover type with low variance (5 – 10), indicating stable dynamics with low variance; region D, where the disturbance is still more frequent, the mature stage is not dominant (< 50% of the landscape) and variance is low (5-10), indicating stable dynamics with low variance; regions C and E, where variance is high (10 – 20) or very high (> 20), respectively, indicating stable dynamics with high variance; and region F, where disturbances are both frequent and large, producing low variance (< 5) and unstable dynamics as the mature stage is no longer the dominant cover type over the duration of the simulation.
