# Supplementary material for: Stochastic disturbance regimes alter patterns of ecosystem variability and recovery
Source: PLoS One. 2020 Mar 9;15(3):e0229927. doi: 10.1371/journal.pone.0229927 (PMC7062255; doi:10.1371/journal.pone.0229927)

Fig S1 Occupancy by successional stage for simulations with deterministic and stochastic disturbance parameters. Scenario a) is deterministic and reproduces the described base model (cf. [30]), b) includes stochasticity in disturbance frequency, c) includes stochasticity in disturbance spatial extent, d) includes stochasticity in disturbance severity, and e) includes stochasticity in all disturbance characteristics: frequency, spatial extent and severity. Parameter values and results for each set of simulations are listed in Table S1.


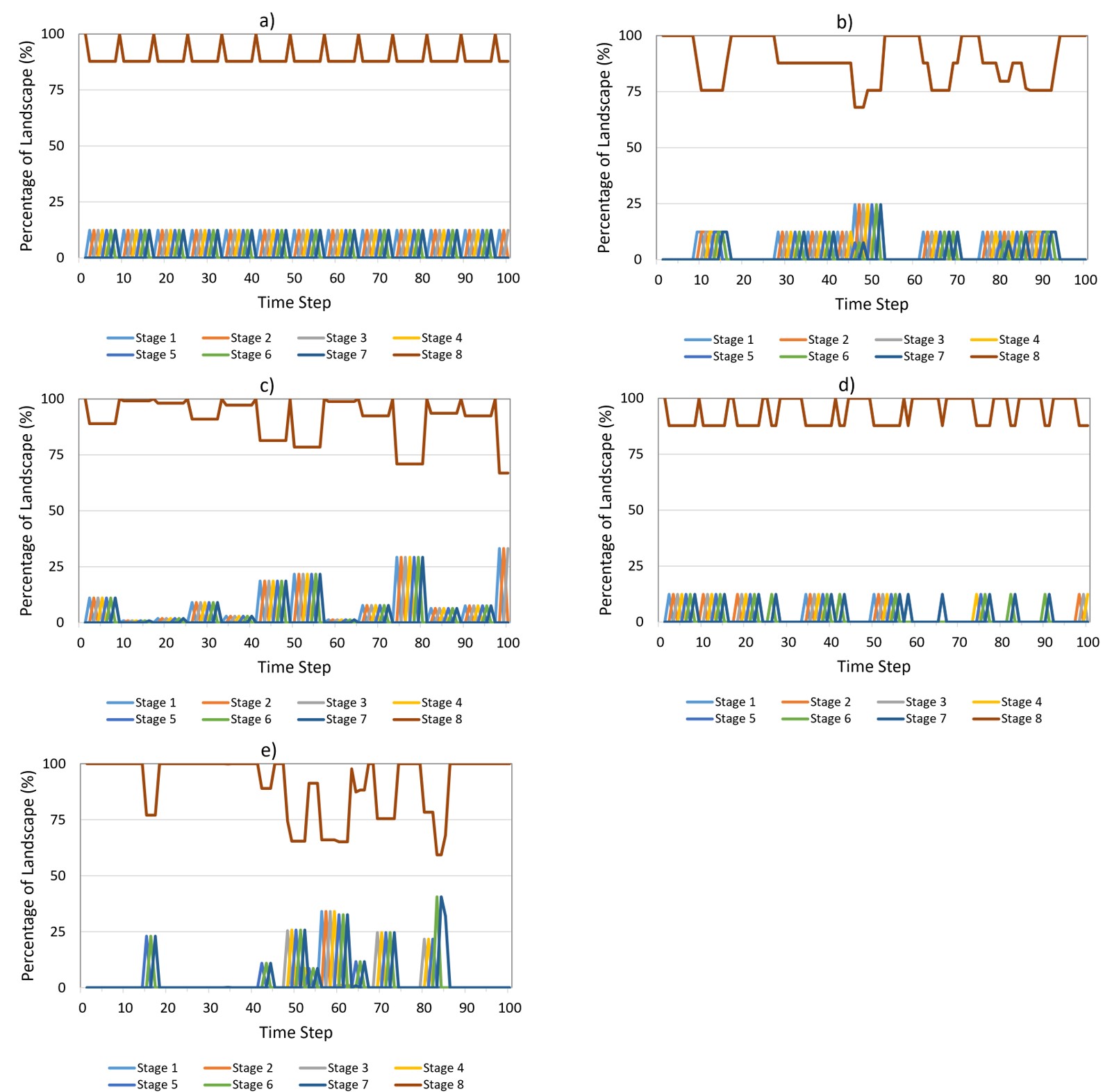

Supplement: S1 Fig — (DOCX) [file pone.0229927.s004.docx]
