# Supplementary material for: Stochastic disturbance regimes alter patterns of ecosystem variability and recovery
Source: PLoS One. 2020 Mar 9;15(3):e0229927. doi: 10.1371/journal.pone.0229927 (PMC7062255; doi:10.1371/journal.pone.0229927)

**Figure S2** **State-space diagrams describing ecosystem dynamics for fully stochastic (i.e., stochastic spatial extent, frequency, and severity) models with 4, 8, or 16 successional stages with respect to temporal and spatial disturbance regime attributes**. In each diagram, we mapped the variance of the proportion of the landscape occupied by the mature successional stage during a simulation of 100,000 time steps. The simulations had mean severities of 1.5, 3.5, and 7.5 for models with 4, 8, and 16 successional stages, respectively. Colors represent the values of variance, and regions with qualitatively different ecosystem dynamics are separated by contour lines.


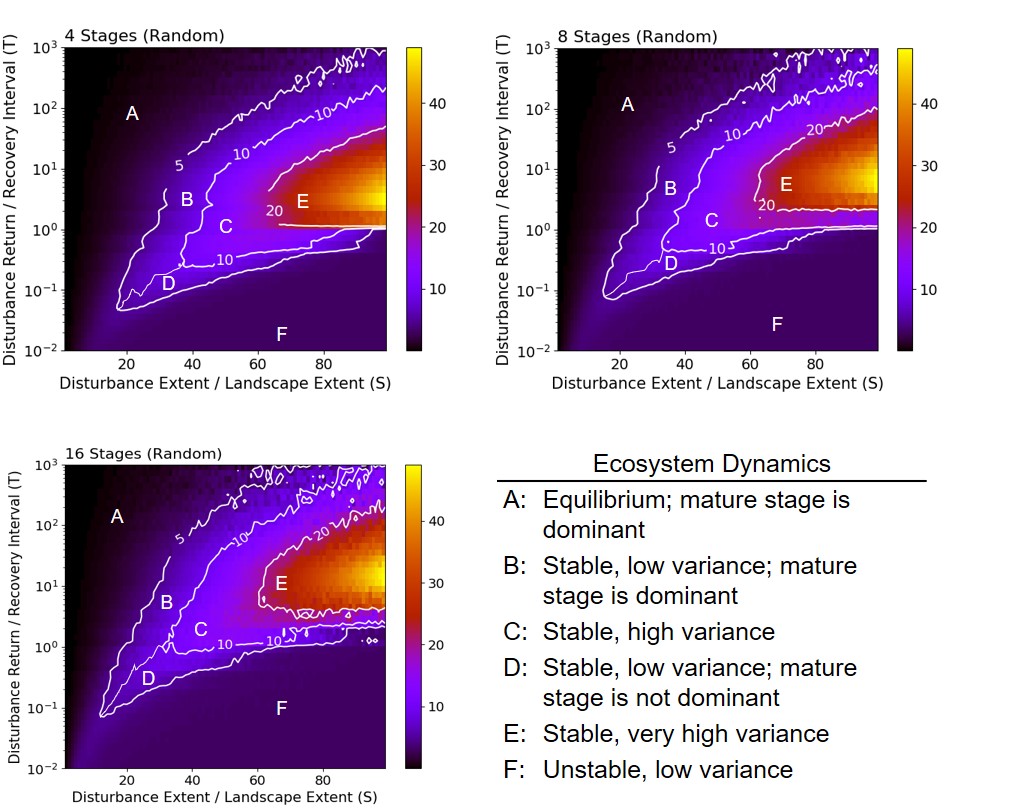

Supplement: S2 Fig — (DOCX) [file pone.0229927.s005.docx]
