# Supplementary material for: Stochastic disturbance regimes alter patterns of ecosystem variability and recovery
Source: PLoS One. 2020 Mar 9;15(3):e0229927. doi: 10.1371/journal.pone.0229927 (PMC7062255; doi:10.1371/journal.pone.0229927)
Supplement: S1 Appendix — (DOCX) [file pone.0229927.s006.docx]

**Appendix S1. Model Validation Results**

Across all simulations, the mean proportion of the landscape covered by the mature successional stage was uniformly high (Table S1, Fig S1). However, variance of the proportion of the landscape occupied by the mature successional stage differed among the simulations. In the deterministic version of the model, each disturbance event sets the disturbed locations to the lowest successional stage and at subsequent time steps the landscape recovers through each of the successional stages until it is fully recovered (Fig S1a), resulting in low variance. Under stochastic frequency but deterministic severity and spatial extent, the landscape did not necessarily recover at regular intervals due to potentially clustered disturbance events (Fig S1b). Lacking strict periodicity in the timing of disturbance events, the simulation produced a larger variance for the mature successional stage (Table S1). By contrast, under stochastic disturbance extent and deterministic frequency and severity, we observed the same periodicity in recovery of the mature successional stage as in the deterministic case, but with much greater variation in the spatial extent of the mature stage (Fig S1c). As in the fully deterministic version, the landscape had time to recover before each event, but the increase in spatial variation resulted in a larger variance for the mature successional stage (Table S1).Simulations executed with stochastic severity had lower mean severity than simulations with deterministic severity because individual disturbance events could have low or high severity (Table S1). After disturbance events of lesser severity, the landscape recovered more quickly to the mature successional stage (Fig S1d). As a result, *Vp* was lower than the variance produced by simulations with stochastic frequency or extent, but higher than the variance generated by the deterministic simulation.

Incorporating stochasticity into all the disturbance parameters resulted in the least stable dynamics (Fig S1e). Despite a relatively small number of disturbance events, we found evidence of temporal clustering and variation in both the spatial extent and severity of the disturbances. The variance produced by this simulation was more than triple the deterministic model variance (Table S1). Interestingly, we note that the effect of stochasticity in all disturbance regime attributes was non-additive. The “extra” variance (compared to the deterministic case) generated by simulations with stochastic extent, frequency, or severity, summed to 13.55. However, the “extra” variance for the fully stochastic simulation was 9.14. Thus, the fully stochastic model produced lower variance than would be expected if effects of stochasticity were additive.
