# Supplementary material for: Stochastic disturbance regimes alter patterns of ecosystem variability and recovery
Source: PLoS One. 2020 Mar 9;15(3):e0229927. doi: 10.1371/journal.pone.0229927 (PMC7062255; doi:10.1371/journal.pone.0229927)
Supplement: S2 Appendix — (DOCX) [file pone.0229927.s007.docx]

**Appendix S2.** References for studies cited in Table S3.

1. Coleman RA, Underwood AJ, Benedetti-Cecchi L, Aberg P, Arenas F, Arrontes J, et al. A continental scale evaluation of the role of limpet grazing on rocky shores. Oecologia. 2006;147(3):556-64.

2. Collins SL. Disturbance frequency and community stability in native tallgrass prairie. Am Nat. 2000;155(3):311-25.

3. Houseman GR, Mittelbach GG, Reynolds HL, Gross KL. Perturbations alter community convergence, divergence, and formation of multiple community states. Ecology. 2008;89(8):2172-80.

4. Hsieh CH, Reiss CS, Hunter JR, Beddington JR, May RM, Sugihara G. Fishing elevates variability in the abundance of exploited species. Nature. 2006;443(7113):859-62.

5. Molinos JG, Donohue I. Temporal variability within disturbance events regulates their effects on natural communities. Oecologia. 2011;166(3):795-806.

6. Queiroz N, Lima FP, Ribeiro PA, Pereira SG, Santos AM. Using asymmetrical designs for environmental impact assessment of unplanned disturbances. Hydrobiologia. 2006;555:223-7.

7. Rusak JA, Yan ND, Somers KM, McQueen DJ, Ramcharan CW. Differences in the variability of crustacean zooplankton communities between manipulated and reference lakes. Archiv Fur Hydrobiologie Special Issues Advanced Limnologie. 2001;56:171-86.

8. Ryan JP, Mcmanus MA, Paduan JD, Chavez FP. Phytoplankton thin layers caused by shear in frontal zones of a coastal upwelling system. Mar Ecol Prog Ser. 2008;354:21-34.

9. Stark JS, Riddle MJ, Simpson RD. Human impacts in soft-sediment assemblages at Casey Station, East Antarctica: Spatial variation, taxonomic resolution and data transformation. Austral Ecol. 2003;28(3):287-304.

10. Vargas R. How a hurricane disturbance influences extreme CO2 fluxes and variance in a tropical forest. Environ Res Lett. 2012;7(3):035704.

11. Warwick RM, Clarke KR. Increased variability as a symptom of stress in marine communities. J Exp Mar Biol Ecol. 1993;172(1-2):215-26.

12. Arkle RS, Pilliod DS, Strickler K. Fire, flow and dynamic equilibrium in stream macroinvertebrate communities. Freshwat Biol. 2010;55(2):299-314.

13. Bêche LA, Resh VH. Short-term climatic trends affect the temporal variability of macroinvertebrates in California 'Mediterranean' streams. Freshwat Biol. 2007;52(12):2317-39.

14. Bertocci I, Maggi E, Vaselli S, Benedetti-Cecchi L. Contrasting effects of mean intensity and temporal variation of disturbance on a rocky seashore. Ecology. 2005;86(8):2061-7.

15. Brown BL. Habitat heterogeneity and disturbance influence patterns of community temporal variability in a small temperate stream. Hydrobiologia. 2007;586:93-106.

16. Chapman MG, Underwood AJ, Skilleter GA. Variability at different spatial scales between a subtidal assemblage exposed to the discharge of sewage and 2 control assemblages. J Exp Mar Biol Ecol. 1995;189(1-2):103-22.

17. Cottingham KL, Rusak JA, Leavitt PR. Increased ecosystem variability and reduced predictability following fertilisation: Evidence from palaeolimnology. Ecol Lett. 2000;3(4):340-8.

18. Forrest J, Arnott SE. Variability and predictability in a zooplankton community: The roles of disturbance and dispersal. Ecoscience. 2007;14(2):137-45.

19. Fuhlendorf SD, Harrell WC, Engle DM, Hamilton RG, Davis CA, Leslie DM. Should heterogeneity be the basis for conservation? Grassland bird response to fire and grazing. Ecol Appl. 2006;16(5):1706-16.

20. Mou P, Jones RH, Guo DL, Lister A. Regeneration strategies, disturbance and plant interactions as organizers of vegetation spatial patterns in a pine forest. Landscape Ecol. 2005;20(8):971-87.

21. Navarro L, Ballesteros E, Linares C, Hereu B. Spatial and temporal variability of deep-water algal assemblages in the Northwestern Mediterranean: The effects of an exceptional storm. Estuar Coast Shelf Sci. 2011;95(1):52-8.

22. Reed DC, Raimondi PT, Carr MH, Goldwasser L. The role of dispersal and disturbance in determining spatial heterogeneity in sedentary organisms. Ecology. 2000;81(7):2011-26.

23. Williams MA, Baker WL. Variability of historical forest structure and fire across ponderosa pine landscapes of the Coconino Plateau and south rim of Grand Canyon National Park, Arizona, USA. Landscape Ecol. 2013;28(2):297-310.

24. Liu W, Xu W, Hong J, Wan S. Interannual variability of soil microbial biomass and respiration in responses to topography, annual burning and N addition in a semiarid temperate steppe. Geoderma. 2010;158(3):259-67.

25. Jellyman PG, Booker DJ, McIntosh AR. Quantifying the direct and indirect effects of flow‐related disturbance on stream fish assemblages. Freshwat Biol. 2013;58(12):2614-31.

26. Jiang Y-Z, Cheng J-H, Li S-F. Temporal changes in the fish community resulting from a summer fishing moratorium in the northern East China Sea. Mar Ecol Prog Ser. 2009;387:265-73.

27. Jones SE, Chiu CY, Kratz TK, Wu JT, Shade A, McMahon KD. Typhoons initiate predictable change in aquatic bacterial communities. Limnol Oceanogr. 2008;53(4):1319-26.

28. Kashian DM, Turner MG, Romme WH, Lorimer CG. Variability and convergence in stand structural development on a fire-dominated subalpine landscape. Ecology. 2005;86(3):643-54.

29. Lardicci C, Rossi F, Maltagliati F. Detection of thermal pollution: Variability of benthic communities at two different spatial scales in an area influenced by a coastal power station. Mar Pollut Bull. 1999;38(4):296-303.

30. Micheli F, Cottingham KL, Bascompte J, Bjornstad ON, Eckert GL, Fischer JM, et al. The dual nature of community variability. Oikos. 1999;85(1):161-9.

31. Piazzi L, Balata D, Cinelli F, Benedetti-Cecchi L. Patterns of spatial variability in epiphytes of Posidonia oceanica: Differences between a disturbed and two reference locations. Aquat Bot. 2004;79(4):345-56.

32. Terlizzi A, Scuderi D, Fraschetti S, Anderson MJ. Quantifying effects of pollution on biodiversity: a case study of highly diverse molluscan assemblages in the Mediterranean. Mar Biol. 2005;148(2):293-305.
