# Supplementary material for: Stochastic disturbance regimes alter patterns of ecosystem variability and recovery
Source: PLoS One. 2020 Mar 9;15(3):e0229927. doi: 10.1371/journal.pone.0229927 (PMC7062255; doi:10.1371/journal.pone.0229927)
Supplement: Attachment — Submitted filename: Response to Reviewers_final.docx [file pone.0229927.s009.docx]

Please see our responses below (in blue):

PONE-D-19-25590
Stochastic disturbance regimes improve prediction of ecosystem variability and dynamics
PLOS ONE

Dear Dr. Fraterrigo,

Thank you for submitting your manuscript to PLOS ONE. After careful consideration, we feel that it has merit but does not fully meet PLOS ONE’s publication criteria as it currently stands. Therefore, we invite you to submit a revised version of the manuscript that addresses the points raised during the review process.

You manuscript has been evaluated by three expert reviewers and myself. Both Reviewer #1 and #2 only raised minor points about your study, whereas Reviewer #3 has more important concerns about your study. I partially agree with Reviewer #3. One the one hand, I understand that your aim is to introduce stochasticity on some parameters that Turner's original work considers deterministic and in that sense your study provides a sufficiently novel contribution to deserve publication in PLOS One (it is not revisit of Turner's work). One the other hand, I think that some parts of the manuscript, such as the heading of the section in which you revisit Turner's model, could be modified to make this distinction as clear throughout the manuscript as it is in the Introduction. In that sense, Reviewer #3 provides very detailed suggestions. Because of this, I am inviting you to resubmit a Minor Revision of the manuscript, but I am planning to send the manuscript back to Reviewer #3 to ensure that the main four points raised in his/her report have been fully addressed.

We would appreciate receiving your revised manuscript by Dec 06 2019 11:59PM. When you are ready to submit your revision, log on to <https://www.editorialmanager.com/pone/> and select the 'Submissions Needing Revision' folder to locate your manuscript file.

If you would like to make changes to your financial disclosure, please include your updated statement in your cover letter.

To enhance the reproducibility of your results, we recommend that if applicable you deposit your laboratory protocols in protocols.io, where a protocol can be assigned its own identifier (DOI) such that it can be cited independently in the future. For instructions see: <http://journals.plos.org/plosone/s/submission-guidelines#loc-laboratory-protocols>

Please include the following items when submitting your revised manuscript:

- A rebuttal letter that responds to each point raised by the academic editor and reviewer(s). This letter should be uploaded as separate file and labeled 'Response to Reviewers'.
- A marked-up copy of your manuscript that highlights changes made to the original version. This file should be uploaded as separate file and labeled 'Revised Manuscript with Track Changes'.
- An unmarked version of your revised paper without tracked changes. This file should be uploaded as separate file and labeled 'Manuscript'.

Please note while forming your response, if your article is accepted, you may have the opportunity to make the peer review history publicly available. The record will include editor decision letters (with reviews) and your responses to reviewer comments. If eligible, we will contact you to opt in or out.

We look forward to receiving your revised manuscript.

Kind regards,

Ricardo Martinez-Garcia
Academic Editor
PLOS ONE

**Journal Requirements:**

1. When submitting your revision, we need you to address these additional requirements.

Please ensure that your manuscript meets PLOS ONE's style requirements, including those for file naming. The PLOS ONE style templates can be found at
<http://www.journals.plos.org/plosone/s/file?id=wjVg/PLOSOne_formatting_sample_main_body.pdf> and <http://www.journals.plos.org/plosone/s/file?id=ba62/PLOSOne_formatting_sample_title_authors_affiliations.pdf>

**Comments to the Author**

1. Is the manuscript technically sound, and do the data support the conclusions?

The manuscript must describe a technically sound piece of scientific research with data that supports the conclusions. Experiments must have been conducted rigorously, with appropriate controls, replication, and sample sizes. The conclusions must be drawn appropriately based on the data presented.

Reviewer #1: Yes

Reviewer #2: Yes

Reviewer #3: Partly

2. Has the statistical analysis been performed appropriately and rigorously?

Reviewer #1: Yes

Reviewer #2: Yes

Reviewer #3: Yes

3. Have the authors made all data underlying the findings in their manuscript fully available?

The [PLOS Data policy](http://www.plosone.org/static/policies.action#sharing) requires authors to make all data underlying the findings described in their manuscript fully available without restriction, with rare exception (please refer to the Data Availability Statement in the manuscript PDF file). The data should be provided as part of the manuscript or its supporting information, or deposited to a public repository. For example, in addition to summary statistics, the data points behind means, medians and variance measures should be available. If there are restrictions on publicly sharing data—e.g. participant privacy or use of data from a third party—those must be specified.

Reviewer #1: Yes

Reviewer #2: Yes

Reviewer #3: Yes

4. Is the manuscript presented in an intelligible fashion and written in standard English?

PLOS ONE does not copyedit accepted manuscripts, so the language in submitted articles must be clear, correct, and unambiguous. Any typographical or grammatical errors should be corrected at revision, so please note any specific errors here.

Reviewer #1: Yes

Reviewer #2: Yes

Reviewer #3: Yes

5. Review Comments to the Author

Please use the space provided to explain your answers to the questions above. You may also include additional comments for the author, including concerns about dual publication, research ethics, or publication ethics. (Please upload your review as an attachment if it exceeds 20,000 characters)

**Reviewer #1:** The authors present an investigation of an extension of the nonequilibrium landscape model by Turner et al. [20]. The model assumes that disturbances on the landscape have stochastic features (occurrence, size, etc...) mimicking realistic conditions. Comparing the simulation results with experimental data, the authors find a good agreement when accounting for stochasticity, in contrast with a deterministic (effective equivalent) model, which fails to predict landscape properties in many cases.

The manuscript is well written and the results are sound and well explained. However, I have minor concerns about the current version as I detail below.

1. The authors strongly emphasize that accounting for stochasticity improves prediction of the variability classification. I understand the point, but it is natural that making the model more complex would increase your predictability. This would occur for any pertinent generalization of the Turner's model. The main message is that stochastic regimes significantly impact on ecosystem variability classification. Of course, when considering it, you increase your predictability. Furthermore, the claim that 'stochasticity increases prediction' gives the idea that a stochastic resonance phenomenon is in place, i.e. stochasticity is enhancing a certain feature of the system that you are trying to measure. So, I would suggest the authors to review this terminology used in the manuscript, being careful in order to allow the results to reach with more clarity a broader audience.

We see the reviewer’s point and agree that the main message is that stochastic disturbance regimes significantly alter patterns and classification of ecosystem variability and resultant recovery dynamics. We have revised the title, abstract, introduction, results and discussion sections to emphasize that message and deemphasize the importance of incorporating stochasticity for prediction.

2. Simple models indeed can help understand the behavior of ecosystems. But, since the proposed one assumes non-interacting units, two points come to my mind. a) I don't see how external perturbations can affect the ecosystem dynamics if the entities are non-interacting (e.g. Ising model). In my point of view, despite the fact that the variability regimes are being affected, the dynamics itself is the same (system response time properties, for instance). So, I suggest the authors to review the statement that stochastic regimes affect ecosystem dynamics (this is related to comment 1), verifying if it is really applicable in the present context. b) Analytical considerations to support the numerical results seem to be possible. In this sense, I missed basic (mean-field) relations (I would guess that the contour lines in Fig.2 are given by T = const. x S, which with y-log scale cannot be automatically understood). Besides this, it seems that a complete theory for the present model, or for other versions, could be developed, or might be already present in the literature. So, including a discussion or references about this aspect might be helpful to others understand the development on the theoretical side.

We believe this concern stems from the reviewer defining ecosystem dynamics differently than we do. Ecosystem dynamics can be defined in terms of composition or structure (i.e., persistence or constancy; Holling 1973). In our study, we define dynamics with respect to structure, focusing on fluctuations in the amount of the mature successional stage, as stated explicitly in the manuscript (line 220-222). Given this, we maintain that altered variability regimes can be interpreted as altered ecosystem dynamics.

While we appreciate the reviewer’s point about the need for a tractable analytical solution, neither myself nor my co-authors possess the skills to find a mathematical solution to the model. Nonetheless, we addressed this comment by adding text to explain why the type of computational model we use is useful in ecological studies (lines 77-83)

Bormann, F. H., and G. E. Likens. 1979. Pattern and process in a forested ecosystem: disturbance, development and the steady state based on the Hubbard Brook ecosystem study. Springer-Verlag, New York.

Botkin, D. B., and M. J. Sobel. 1975. Stability in time-varying ecosystems. American Naturalist 109:625-646.

Halpern, B. S., E. T. Borer, E. W. Seabloom, and J. B. Shurin. 2005. Predator effects on herbivore and plant stability. Ecology Letters 8:189-194.

Holling, C. S. 1973. Resilience and stability of ecological systems. Annual Review of Ecology, Evolution, and Systematics 4:1-23.

Loucks, O. L. 1970. Evolution of diversity, efficiency, and community stability. American Zoologist 10:17-&.

Sprugel, D. G. 1976. Dynamic structure of wave-regenerated *Abies balsamea* forests in northeastern United States. Journal of Ecology 64:889-+.

**Reviewer #2:** "Stochastic disturbance regimes improve prediction of ecosystem variability and dynamics" is a very interesting and well-written work.
The study focuses on an important contemporary problem and highlights the importance of considering disturbance fluctuations for a better understanding of the response of actual ecological systems.
Contents are very clear, well organized and exposed. The analysis is done carefully.

Comments:

1. Although already said that extensive analysis is left for future studies, I think it is important a longer discussion about the robustness of the results to other parameter values and degrees of stochasticity.

The aspects of disturbance that we explore are arguably the most important attributes (White and Jentsch 2001) and we feel that extrapolation beyond the current parameters explored may be too speculative to include. We thus restrict ourselves to discussion of the parameter values robustly examined in the paper. That said, we emphasize that exploring other parameter values and degrees of stochasticity is an important next step for improving understanding of how stochasticity affects patterns of ecosystem variability and recovery.

2. The considered dynamics probably experience transient and stationary regimes. For most of the analysis, 10000 time steps are considered, probably reaching the stationary state. Results would then be averaged over transient and stationary regimes, which have different behaviors. I think it is important to discuss this point and clarify its validity.

We performed simulations for 100,000 time steps, not 10,000 time steps. This was stated correctly in the text, so the reviewer may have missed it. Models generally stabilized in < 100 time steps, so any influence of the transient phase on calculated metrics of ecosystem dynamics (i.e., mean and variance) were minimal. We added text to the manuscript to address this comment (lines 242-244).

3. Concerning the previous point, in section (i) 100 time steps are considered, showing the transient dynamics. I propose to discuss a case with longer times.

We explicitly chose to run the simulation for 100 time steps so that we could validate the model and visually investigate recovery dynamics across successional stages. We have moved the model validation results to the supplement and now focus the paper on the simulations in which we run the model for 100,000 time steps.


Minor comments:

1. In line 122, "spatial extent" has not been defined previously.

We changed “spatial extent” to “size” to reduce ambiguity. The term “spatial extent” is defined in the following paragraph.

2. In line 134, it is not clear how frequency is implemented.

To address this concern, we clarified how frequency is implemented in the model (137-139). We also added text that directs readers to the section “Stochastic Frequency.” An example of how frequency is implemented is provided in that section (line 162-167).

3. In line 143, the concept of "severity" remains slightly unclear.

To clarify, we added the phrase “impact on the ecosystem” parenthetically. (line 147)

4. In line 176, I think it should be <=2.

The statement is correct as written. We define disturbance severity with respect to the successional stage of a cell. A high severity disturbance would set back the successional stage of a cell to a low value, representing an early successional stage. In contrast, a low severity disturbance would set back the successional stage of a cell to a higher value, representing a later successional stage. A cell at a successional stage of 2 would already be at an early successional stage and would not “experience” disturbance. We added text to clarify how we implemented stochastic disturbance severity (begins on line 175).

5. In line 184, "width and height" is sometimes changed to "width and length". One of them should be chosen. Maybe "width and length" would be more appropriate for 2D systems.

We changed “height” to “length” throughout the manuscript.

6. Defined regions are slightly confusing. In particular, "stable" refers to both >50% and <50% of mature stage dominance. For example, In Fig. 3 regimes B and D are both described by "stable, low variance".

We followed the approach of Turner et al. to define these regions. However, we can see the reviewer’s point about the definitions being slightly confusing. We have revised the text and the legends in these figures (now Figs 5 and 6) to increase clarity.

7. In line 276, "Comparison WITH OF published..."

We removed the word “with” from the section heading.

8. In line 356, "spatial extent" should be "mature successional stage"?

We meant the spatial extent of the mature successional stage. We revised this sentence to increase clarity.

9. In line 379, as below, the % of the expanded region can be indicated.

We have added this information to the Results (lines 334 and 351).

10. In line 417, clarify that it is the overlaying of Fig. 3?

Done.

11. In Fig. 1, stages 2,3,4,5,6 are not well distinguished with the selected dashed lines.

We replotted this figure in color. This is now Fig S1 because we moved the model validation results to the supplement.

12. In Fig.2, yellow indicates 100% and black 0% instead of >50% and <50%, respectively.

We revised the figure caption accordingly. This is now Fig 1.

13. In Fig.2, the deterministic case is not shown for comparison.

We have added a panel showing the deterministic case for comparison. This is now Fig 1a.

14. In Fig. 2, 3, S1 and S2, colorbox values are not defined in the captions.

We have added this information to the Fig. 1 caption.

15. In Fig. 2, 3, S1 and S2, colorbox values are difficult to read because of the small font size.

For all the figures, we have enlarged the font size to increase legibility.

16. The analysis of Fig 4. could be done for other parameters. Maybe it can be discussed what happens for the rest of the parameters.

We do not understand what other parameters the reviewer would like for us to analyze. Figure 4 (which is now Fig 6) compares the simulated dynamics for the deterministic case to those produced by introducing stochasticity in each of the disturbance parameters considered by the model: (a) frequency, (b) extent, (c) severity, and (d) a stochasticity in all three parameters. All possible combinations are presented in the figure.

**Reviewer #3:** The manuscript by Fraterrigo et al. looks at how various forms of stochasticity of disturbances affects ecosystem dynamics using a simple theoretical methodology, and use previously published work to test how various ecosystems conform to their model. I believe that the goal of the authors is a very important one, namely better understanding of how stochasticity impacts ecosystem dynamics and predictions related to them, and trying to test this on data from various ecosystems. However, I do not think that the approach the authors took to do this is the most appropriate, and I believe it has several important flaws.

These main issues are:

1) There is some discrepancy in what do the authors aim to achieve in this paper? Is it a revisit of the classic Turner paper, or a test of the role of variability in various disturbance parameters? The way the analysis is done and presented it seems that this is a revisit of the Turner paper (e.g. Fig. 1 and its specific parameters that are the same as in the Turner paper), while the discussion and general claim of the paper is that this is an attempt to understand the role of variability. I think the authors need to choose one of these. Also, assuming that they want the second option, then a better discussion or analysis on the role of the different stochasticity sources, and in particular what happens to their combination, is quite necessary. See also points below: 13, 17 ,18, 20, 21, 25, 26, 32, 37.
The primary goal of the paper is to understand how stochasticity in disturbance attributes affects ecosystem variability and resultant ecosystem dynamics. However, we contend that adequately addressing this topic necessitates a revisit of the Turner study to some degree. The Turner et al. study made a seminal contribution concerning the role of disturbance in altering ecosystem dynamics and continues to be highly influential (it has been cited 552 times, including as a textbook example). In this respect, the Turner study provides valuable context for our study. Nevertheless, we can see the reviewer’s point and have addressed it by revising the manuscript to de-emphasize the comparison between our findings and those of Turner’s. Specifically, we moved the results pertaining to model validation (where we directly compare using the specific parameters from the Turner paper) to the supplement. We have also increased the discussion on the role of the different stochasticity sources, separately and in combination. See below for a detailed description of the changes made.

2) Much of the assessment/comparison is based on the arbitrary distinction to 6 regions (A-F). Saying that something is significant because it changes the classification between these 6 regions, without explaining how they are qualitatively different or why this classification is objective, is flawed. I believe that this is an issue of the mixture between "two papers" I mention in my previous point. See also points below: 16, 24, 25, 28.

While we appreciate the comment, we disagree with the reviewer on these points. First, the 6 regions are not arbitrary. They were defined based on the numeric results of simulations. Thus, while they describe qualitative differences, the regions are derived from quantitative patterns. Our approach to defining these regions was explained in the original version of the manuscript, but we added additional text to increase clarity (lines 248-250). Specifically, we indicate that these regions represent the range of observed or theoretically predicted dynamics, including steady state dynamics (Sprugel 1976, Bormann and Likens 1979), stable dynamics with repeating recovery sequences characterized by low or high response fluctuations (Loucks 1970, Botkin and Sobel 1975), and unstable dynamics (Halpern et al. 2005). To address the concern, we reorganized the results to emphasize the quantitative patterns while retaining the qualitative results (see Results section from line 266 on). The figure showing the quantitative differences in variance is now in the main body of the manuscript as Fig 3 (was Fig S1).

3) The comparison to data is problematic, and seems very skewed/arbitrary to authors choices of how to classify a given system. The way the authors chose to classify each system certainly needs to be better explained, but it also seems that it is quite arbitrary, and therefore why we should believe that this classification and its repercussions makes sense needs to be explained. Regardless, given this issue, and what seems like a problematic data set, it seems that the main claim of the paper, that this addition of stochasticity improves predictions, needs to be toned down. See also points below: 14, 33.

We agree that we can improve the justification for and explanation of our approach. Our approach to classifying variability patterns was designed to allow comparison across ecosystems and disturbances which can vary greatly in spatial and temporal scales. We used a similar approach to classify variability in our previous work and found it useful for highlighting unknowns and informing future research directions (Fraterrigo and Rusak, 2008 Ecol. Lett.). We have revised the methods accordingly. We have toned down the claim concerning prediction. See our response to Review 1’s comment #1. We have also indicated the problems associated with the comparison component of our paper in a penultimate discussion paragraph that we added to explain caveats inherent in this analysis.

I would like to clarify some things regarding these 3 points. First, I do appreciate the choice of using a simple model, and focusing on qualitative information to test this on data. While I have some issues with the choice of model taken and its applicability, this is not in itself my main concern, but rather the discrepancy between the proposed goal of understanding stochasticity and the actual work done, which in practice mainly focuses on this models's specific results. I also appreciate that finding and collecting the data on various systems is not a simple task, and that most likely there is not enough data out there currently to preform a more detailed analysis. However, if this is the case, then this issue should be better addressed (by explicitly stating the limited power of the test preformed, toning down the strong claim (e.g. title) the authors make, and perhaps mentioning in the discussion that better data is needed).

We appreciate the clarification and encouragement and agree about the need to refocus the ms and explain better the limitations of the modeling approach. We have made several changes to address this comment along the lines suggested by the reviewer. First, we revised the title. Second, we toned down the claim about improving prediction throughout the manuscript. Third, we inserted text relating to the limited power of the tests performed (see previous comment). Finally, we acknowledge the need for better data (lines 591-601)

I list below my other comments and issues, mostly organized by line numbers. While they are not major on their own, many of them make up the main issues I raised above.

4) I think in the introduction some more context is needed on previous work. In particular other works looked at similar issues of disturbance properties (e.g. Miller 2011 PNAS, Miller 2012 Eco Res), many studies looked at other system properties that are ignored here in relation to disturbances (e.g. Leibold 2004 Ecol. Lett. on dispersal, Zelnik 2019 Frontiers on local dynamics), and other models have been used to look at similar issues (e.g. Moloney & Levin 1996 Ecology, McCabe&Dietz 2019 Frontiers).

We revised this section to introduce more context concerning disturbance effects on recovery dynamics. Specifically, we highlight the role of biotic factors, including species life history, competitive ability, and dispersal capacity, citing several of the papers suggested by the reviewer (lines 72-73). We also discuss other modeling efforts aimed at improving understanding of disturbance effects (lines 74-76).

5) lines 59-71 have some strange phrasings of the topic - line 59: "the response of ecosystem variability", line 61: "disturbance-driven changes in the variability of a response can indicate differences in", line 71: " altered ecosystem variability". I guess you mean to talk about about "changes in variability", how they might matter or inform us, etc. But this is currently unclear.

We revised these sentences to increase clarity.

6) lines 73-75: It is not true that there are 3 relevant properties of disturbances (frequency, severity & extent). For instance, species composition (i.e. how different species are sensitive to a given disturbance) might matter significantly, see for instance Arnoldi et al. 2018 JTB.
We agree that other disturbance properties can affect recovery dynamics. In the original version of the ms, we stated that biotic and physical factors can modulate disturbance effects and recovery. We have expanded on this point. We have also stated that other disturbance regime properties can affect recovery dynamics (line 75).

7) lines 83-89 - The purpose of this text is not clear, and seems like it is mostly unnecessary.

We have revised this section and deleted the text that was unclear.

8) line 152 - The authors themselves defined disturbance frequency in a previous publication "It is widely recognized that disturbance frequency, defined as the mean number of events per time period...", in a way that is not consistent with talking about "stochastic frequency" versus "constant frequency", since both would be called frequency in any case. It would be better to talk about return interval for this definition, if not elsewhere in the manuscript.

Yes, we acknowledge that terminology is a problem. However, for a given disturbance regime, even return interval is typically defined as the average RI. Moreover, as we stated, frequency and RI are directly related, so we do not agree that using the term RI in place of frequency will help to resolve this issue. We contend that clearly defining what we mean by “stochastic frequency” is the best way to avoid confusion.

9) lines 153-162 this seems ill defined. Are you really always having 16 events? In this case, you should better describe your randomization process (is this a point process?)? Or do you just have 16 disturbances on average? (which would make more sense)

We state that 16 events is a simple example used for comparison with the original deterministic model. We have adjusted and added to the wording in this section in order to clarify confusion about the process of selecting randomly frequent events (lines 162-167).

10) lines 196-200 seem counter productive - there is stochasticity in all these processes (even those controlled by humans), it is more the question of how much variation exists, and therefore, how much it might matter that they are stochastic. Trying to untangle the roles of these different forms of stochasticity, and their combination, is certainly a good idea, but the logic of doing so should be stated clearly.
We revised this section as suggested.

11) lines 207-208 and lines 249-250 give very different runtime numbers for simulations - why?

We have two different goals. The first is to validate the model and visually interpret the recovery dynamics under deterministic and stochastic parameterizations. The second is to generalize the results for a wide range of S and T values. For that, we need much longer runtimes to calculate a statistically robust mean and variance. To avoid confusion, we have moved all the results pertaining to model validation to the supplement.

12) line 286 - what does it mean that "We focus on variance"? If it is simply that the mean doesn't change much between your 4 models, why not say that explicitly?

We revised this sentence as suggested.

13) lines 292-304 - is there anything different between what you did for the model runs here and for the T-S parameter space? I guess not, and if so, this whole explanation is cumbersome and unnecessary, since as I understand it, you basically estimated where the different systems (from publications) are on the T-S parameter space, given values of S and T for each system.

The approach here differs from what was done for the S-T parameter space in that we used the actual values of spatial extent, frequency, and severity reported in the studies to parameterize the deterministic and stochastic models, and directly calculate mean and variance. We then used the mean and variance to classify the patterns following previously explained approach. We provided a detailed explanation because we felt it was important to be explicit about the approach to avoid confusion.

14) line 311-313 - How is this classification done? This is a critical point, to which you basically give no information, which is particularly noteworthy considering you give plenty of (often too much) information about the specific model choices and technicalities.

We have addressed this comment above (see also our response to #2).

15) lines 319-320 are strange. You say you measure S and T to "facilitate interpretation"? But, without estimating S and T, no comparison can be done - is that not so?

Because we calculated the mean and variance directly from the model simulations and used these data to classify the dynamics, we could determine data-model agreement for each individual study without determining S and T. Our goal in estimating S and T was to facilitate comparison across studies. We have revised the text to increase clarity.

16) lines 331-334, at least on their own, seem unsubstantiated. Why are differences in variance levels indicating "distinct ecosystem dynamics". No qualitative shift, if one exists, is mentioned. Also, why were this specific parameter set chosen, besides the fact that it was used in the original Turner paper? How general is the result that averages do not change much, but variances do?

We deleted “distinct ecosystem dynamics” from the sentence. The specific parameter set was chosen so that we could validate the model and visually interpret how the introduction of stochasticity altered recovery dynamics. The generalized results are provided through the analysis of S and T values.

17) lines 336-341 - Overall I would say that Fig.1 chooses parameter that are not very representative. Given this specific frequency choice, disturbances cannot interact, but this is not an obvious choice. For any frequency that is higher interactions are likely to occur, and this should be mentioned.

Yes, this is true. As stated above, we have tried to clarify that the goal here was to validate the model rather than offer general insights. These results have been moved to the supplement.

18) lines 346 and 352, "indicating high ecosystem stability" and "indicating reduced ecosystem stability" - what is meant by stability here? If it is the lack of variability, then this is correct, except there's no need for "indicating" - it is a fact. If it is something else, then that needs to be better explained.

We deleted these phrases.

19) lines 347-364 are mostly stating things that are quite evident. I think they can be shortened or clarified.

We shorted this section considerably and moved it to the supplement.

20) lines 368-369 - but how much is the variance compared to the "extra" variance from all 3 separately?
 This is an interesting point. If we sum the “extra” variance generated by simulations with stochastic extent, frequency, or severity, we get 13.55. The “extra” variance for the fully stochastic simulation is 9.14. So, the effects of stochasticity are not additive, but rather synergistic such that a fully stochastic model produces lower than expected variance and greater stability. We added this information to Appendix S1, which described the model validation results. Furthermore, estimates of mean and variance are presented in Table S1 (previously Table 1). We added text that directs the reader to the table.

21) lines 371-377 are again almost trivial. A multiplication of T*S would tell me the baseline values I would expect for the mature stage. What would be interesting to know is how it drifted from this baseline.

To address this comment, we shortened the existing text and added text and a figure (Fig 2) showing how the coverage of the mature successional stage differed with stochastic parameters compared to deterministic parameters (lines 349-356).

22) lines 378-379 a bit vague, and not something that is apparent from the figure itself. How much actually changed. And can it be accounted for by "adding" the 3 separate components?

We addressed this comment above. See Reviewer 2, comment #9, and Reviewer 3, comment #21.

23) lines 386-389 appear to be wrong. Did you mean that the areas below and above 50% are separated by the white line?

Yes, we fixed this mistake. See Reviewer 2, comment #6

24) lines 391-396 Amplified by how much? Perhaps a map of this would be more illuminating then some of these other measures that use arbitrary classifications.

We addressed this comment along the lines suggested. See Figs 3 and 4 which shows the quantitative changes in variance resulting from stochastic model configurations compared to deterministic model configurations. Changes are characterized as the difference in Vp between stochastic and deterministic simulations.

25) lines 397-399 comparing the fully stochastic scenario, with extent of frequency scenarios, region C grows less for the fully stochastic, but region E grows more. Is this because they are related to each other, or is something else happening?
That is an interesting observation. Fig 6 suggests that this happened because the variance increased substantially under fully stochastic parameterizations, so a large portion of the state space that was classified as C, D, and F is now classified as region E. We have incorporated these results.

26) lines 402-407 tell us things we could see from before, which is fine. But what about an explanation of what is happening? In particular, is the severity low variance related to the specific choice where it can only be lower for the stochastic scenario? What if you compared a more appropriate scenario?

We added text and a figure to explain further what is happening with respect to stochastic severity (lines 465-470, Fig S2).

27) line 413 - "major transitions in ecosystem dynamics", again the claim that this represents major transitions seems unsubstantiated.

We toned this down and explained above how we arrived at the different classes.

28) lines 418-420 - It seems that you are saying that near the "boundaries" between your regions (A-F), there are more changes between regions due to addition of different sources of stochasticity. This is to a large extent a form of tautology, since changes near an edge of a region are more likely to lead to a different region due to geometric considerations alone. Or did you mean something else?

We revised this section, highlighting that changes in qualitative states occurred most often when disturbance regimes had intermediate frequencies (lines 445-449).

29) lines 427-429 - this should be given in context. It is an artifact of the parameter space, e.g. stochasticity means less when disturbances occur very infrequently (no interactions between disturbances) or when they are very frequent (system is almost always at the lowest stage).

We have added context along the lines suggested (lines 369-373).

30) lines 439-441 - how is this different than before? Throughout the paper you test 4 different stochastic models, and it seems that here as well. So what is different about this? Also, it is not clear what parameter you used here to get Fig. 5.

This has been clarified in the manuscript. Figure 4 (previously Fig 5) offers a cross-sectional view of Figure 3 in order to isolate the changes in variance over different values of frequency. We have also re-labelled the figure in order to increase clarity.

31) lines 442-445, I guess you meant "f" instead of "T"?

We meant 1/f, disturbance return interval. We have corrected this and axis title in the figure.

32) lines 439-451 - key insights are missing here. The fact that for T>8 (small f) you get a larger difference between the deterministic and stochastic models is not very surprising, considering that for the deterministic model you cannot get interactions of disturbances, but construction. When T<8 (high f) this changes, and things become more comparable. Interestingly, this is exactly when the extent-only model has larger variance than others, which is consistent with the fact that it doesn't have interactions for T>8 (i.e., the extent-only model "should" get larger values overall, but for T>8 this is not seen). Also, why is the peak around T=1? Is that a general result?

We agree and have addressed this comment by expanding the description of the role of disturbance return interval (1/f) along the lines suggested by the reviewer (lines 385-412).

33) lines 467-476 - over 60% of papers were with disturbance size=1. This seems quite problematic for the analysis, and at the very least should be mentioned.

We added a sentence noting that over 60% of the had S = 1 (line 475). We have also acknowledged this limitation in the Discussion (lines 591-601).

34) lines 502-505 - are you trying to say that stochasticity matters, and that variability and stochasticity are related? That alone seems redundant. If you are saying something else, it should be better clarified.
We revised the paragraph and deleted that sentence.

35) lines 506-519 seem to just repeat results and conclusions from the original Turner paper. And lines 519-527 are quite strange and seem out of place. In particular, talking about dispersal while it is not mentioned at all in the whole paper, would only make sense if this was to discuss how adding dispersal would affect the results - and this is not what the authors do here.

We revised the discussion, making every effort to highlight the novel findings while not over-reaching. We also removed the section about dispersal and co-existence, in line with the reviewer’s suggestion.

36) lines 543-550 seem to try and make several points, which seem either problematic or that I could not follow properly. In the first sentence you say "in accordance" - of what to what? Are you just saying that the addition of variance due to stochasticity in frequency and extent lead to larger regions ("stable, high or very high variance")? Why does this "suggest" that there is increased fluctuations? And regardless, why not check that explicitly? Also, why did "ecosystem dynamics remained stable..."? Stable in what sense? And how does this indicate that stochasticity does not need to drive systems to alternative states? Certainly, that statement as is (I would rephrase it as "stochasticity will not always drive systems to collapse in finite time") is true, but it is also widely known, and rather, any divergence from it would be surprising. Finally, one point that I suppose you tried to make, but I am not at all sure, is that

We revised this paragraph to increase clarity. Specifically, we deleted the phrase starting with “in accordance”, changed “suggests” to “because”, and explained what we meant by “remained stable.” We also revised the statement about alternative states as suggested.

37) lines 562-572 point out to a major modeling problem, which is that the comparison of deterministic vs. stochastic severity of disturbances was not done properly. The last sentence calls on more research in this direction, but I think that the minimal thing you needed to do was test this directly, even if in a limited fashion (e.g. compare deterministic model with severity 8 to a stochastic one where severity is randomly chosen between 15 an 1)

In fact, we did what the reviewer recommends in the original version. I.e., to explore the sensitivity of the model to the number of successional stages and differences in mean severity, we repeated the simulations using a model with 4 and 16 successional stages. Results are presented in Fig S2 (previously Fig S3). We revised the text to clarify this point (lines 573-577).

38) lines 586-606 - I think it is here that you need to mention the effect of dispersal, that your model does not account for, similarly to how you do this with issues of internal dynamics.

We have noted that dispersal is unaccounted for (line 605) and have expanded our explanation of the model’s behavior in the absence of an explicit treatment of internal dynamics.

39) Figure S1 does not use colors, and the color axis is not the same for all panels, making visual comparisons difficult.

We fixed this. All the figures are now in color and the color axes match.

40) "Turner et al. [20]" is used many many times throughout the manuscript. This can often be shortened, e.g. "deterministic model"

We made the suggested edit.

41) line 71 - "Disturbance" should be "A disturbance" or "Disturbances", no?

We made the suggested edit.

42) line 253 - typo: "produced by using"

We have corrected this typo.

43) line 276 - typo: "Comparison with of published"

 We have corrected this typo.

6. PLOS authors have the option to publish the peer review history of their article ([what does this mean?](https://journals.plos.org/plosone/s/editorial-and-peer-review-process#loc-peer-review-history)). If published, this will include your full peer review and any attached files.


If you choose “no”, your identity will remain anonymous but your review may still be made public.


**Do you want your identity to be public for this peer review?** For information about this choice, including consent withdrawal, please see our [Privacy Policy](https://www.plos.org/privacy-policy).

Reviewer #1: No

Reviewer #2: No

Reviewer #3: Yes: Yuval Zelnik


[NOTE: If reviewer comments were submitted as an attachment file, they will be attached to this email and accessible via the submission site. Please log into your account, locate the manuscript record, and check for the action link "View Attachments". If this link does not appear, there are no attachment files to be viewed.]


While revising your submission, please upload your figure files to the Preflight Analysis and Conversion Engine (PACE) digital diagnostic tool, <https://pacev2.apexcovantage.com/>. PACE helps ensure that figures meet PLOS requirements. To use PACE, you must first register as a user. Registration is free. Then, login and navigate to the UPLOAD tab, where you will find detailed instructions on how to use the tool. If you encounter any issues or have any questions when using PACE, please email us at [figures@plos.org](mailto:figures@plos.org). Please note that Supporting Information files do not need this step.

*In compliance with data protection regulations, you may request that we remove your personal registration details at any time.* [*(Remove my information/details)*](https://www.editorialmanager.com/pone/login.asp?a=r)*. Please contact the publication office if you have any questions.*
